# Impact of different storage conditions with combined use of ethylene blocker on ‘Shalimar’ apple variety

**DOI:** 10.1038/s41598-024-57688-6

**Published:** 2024-04-11

**Authors:** Kartik Khera, Felix Büchele, Rachael Maree Wood, Fabio Rodrigo Thewes, Roger Wagner, Michael Helmut Hagemann, Daniel Alexandre Neuwald

**Affiliations:** 1 Lake of Constance Research Centre for Fruit Cultivation (KOB), Schuhmacherhof 6, Ravensburg, Germany; 2 Department Production Systems of Horticultural Crops, University of Hohenheim, 70593 Stuttgart, Germany; 3 University of Santa Maria, v. Roraima n 9702 1000 Cidade Universitaria, Bairro - Camobi, Santa Maria, Santa Maria, RS 97105-900 Brazil; 4 Horticulture and Product Physiology, Wageningen University and Research, Droevendaalsesteeg 1, 6708 PB Wageningen, The Netherlands

**Keywords:** *Malus domestica* Borkh., Volatile organic compounds, Controlled atmosphere, Dynamic control atmosphere, Regular atmosphere, 1-methylcyclopropene, Plant physiology, Mass spectrometry

## Abstract

This research investigates the impact of storage conditions on the quality and preservation of 'Shalimar' apples, a relatively new cultivar known for its resistance to apple scab and powdery mildew. The study explores the efficacy of different storage techniques such as regular atmosphere (RA), controlled atmosphere (CA), and dynamic controlled atmosphere with CO_2_ Monitoring (DCA-CD), as well as the integration of 1-methylcyclopropene (1-MCP) at different storage temperatures (1 °C and 3 °C). Various fruit quality parameters were monitored under different storage conditions, including firmness, titratable acidity, total soluble solids, background color, respiration, ethylene production, and volatile compounds. The results indicate that the controlled atmosphere (CA) at 1 °C emerges as an efficient method for long-term storage. However, it is noted that CA storage may impact the apple aroma, emphasizing the need for a balance between preservation and consumer acceptability. On the other hand, DCA-CD at variable temperatures (approximately 2.5 °C) offers a promising approach for maintaining fruit quality and a higher concentration of volatile compounds. Integrating 1-MCP enhances firmness, but its impact varies across storage conditions. Principal component analysis (PCA) provides insights into the relationships between storage conditions, fruit quality, and volatile compounds. This study contributes valuable insights into optimizing storage strategies for ‘Shalimar’ apples, addressing sustainability and quality preservation in apple production.

## Introduction

In the production of apples, only a limited percentage of fruit can be immediately sold after the harvest^1^. Therefore, the majority of apples are stored for an extended period to keep fruit available for the market throughout the year and to adapt to the current market situation, avoiding market instability^1^. To avoid post-harvest losses and increase marketability during the apple harvest window, the fruit must be stored for several months^2^. Reducing ethylene metabolism and fruit respiration during storage is necessary to maintain fruit quality^3^. Lowering storage temperatures, using ethylene blockers such as 1-MCP and adjusting the storage atmosphere are some of the most common ways to maintain apple fruit quality during storage^3,4^. A controlled atmosphere (CA) like ultra-low oxygen (ULO) is often used to maintain fruit quality and reduce the incidence of physiological disorders during and after storage^5^. Nearly all recommendations for CA storage regimes are static, which means the CA conditions are permanently fixed from the beginning until the end of storage. However, the fruit metabolism during storage is dynamic and changes according to its physiology. Moreover, storing fruits in CA and applying 1-MCP inhibits apple aroma formation, adversely affecting consumer acceptability^6^. Therefore, an ideal storage environment would change according to the demands or stress of the fruit^7–10^.

DCA can be broadly defined as an arrangement that allows communication between the fruit and the storage facility personnel, allowing the customization of oxygen levels according to the fruit demands during storage^11^. Under DCA, apples are stored at the lowest oxygen limit (LOL) tolerated by fruit before anaerobic metabolism occurs^12,13^. DCA technology is widely preferred over CA, as various studies have shown better maintenance of apple quality^12,14^. However, the LOL of the specific fruit material is affected by pre- and postharvest conditions^15,16^. Therefore, the trial of this technology on different apple varieties is essential for its worldwide adaptation. Efficient monitoring of fruit physiological status is essential for commercial DCA systems. Various technologies are in use to monitor fruit physiological status during storage, such as chlorophyll fluorescence (DCA-CF), ethanol production (DCA-Eth), respiration quotient (DCA-RQ) and carbon dioxide production by fruit (DCA-CD)^12,17,18^. However, a shortcoming of DCA-CF and DCA-Eth is that fruit metabolism is monitored using secondary metabolism^15^. In comparison, DCA-CD and DCA-RQ have an enormous potential of being a commercial success as these methodologies provide a more accurate indication of fruit physiological status and do not require the installation of any additional device^16,18^. For DCA-CD monitoring, the CO_2_ measurement can be performed using the same gas analyzer used to control the atmosphere in a regular static CA /ULO storage room. Furthermore, the measurement of CO_2_ production in the commercial room^4,9^ is not significantly influenced by the tightness of the CA / ULO room (oxygen influx), which is the main issue for the DCA-RQ method. Therefore, the DCA-CD method emerges as a new way to facilitate the implementation of DCA in commercial rooms. Fruits stored in DCA can be stored at higher temperatures, leading to the formation of better aromatic compounds. DCA storage also induces the production of anaerobic metabolites like acetaldehyde, ethanol, and ethyl acetate^19^. Ethanol is known to suppress ethylene action, whereas acetaldehyde and ethyl acetate plays an essential role in inducing favorable aromatic compound.

1-MCP is an effective inhibitor of ethylene action due to its viable binding on the ethylene receptor in the plant tissue^20^. Previous research has shown that 1-MCP prolongs apples' storage life by suppressing the ethylene action, respiration, fruit softening, and loss of titratable acids (TA)^21–25^. This occurs through its binding to ethylene receptors, resulting in the creation of a complex that diminishes the activity of enzymes engaged in ethylene production. Concurrently, 1-MCP also hinders the activity of pectin methylesterase and polygalacturonase, impacting the cell wall's integrity, thereby contributing to the maintenance of fruit firmness^25,26^. The application of this compound is instrumental in maintaining the high quality of apples for prolonged storage, facilitating their export to distant markets^24^. However, the efficacy of 1-MCP largely depends on the variety. Low oxygen storage may also complement the effect of 1-MCP.^27^ found that fruit response to 1-MCP is higher from low oxygen CA (1.2 kPa O_2_ + 1 kPa CO_2_) than in those from standard CA (2.5 kPa O_2_ + 2 kPa CO_2_) at 2 °C. However, other work indicates no further beneficial effect on the storage quality of ‘Empire’, ‘Gala’, and 'Golden Delicious’ apples from combining CA at low O_2_ with 1-MCP^27^. Henceforth, it is essential to check the efficiency of 1-MCP in different storage conditions for different apple varieties.

The relatively new apple cultivar ‘Shalimar’ is a cross between ‘Topaz’ and ‘Golden Delicious’^28^. ‘Shalimar’ is known to be resistant to apple scab and powdery mildew; thus, fewer pesticides are needed for its production compared to other cultivars. ‘Shalimar’ has a well-balanced sugar and acid content^28^. Due to its natural resistance to apple scab and powdery mildew, this cultivar marks a notable advancement in sustainable fruit production. It diminishes the need for chemical pesticides, promoting environmentally friendly farming practices. However, as it is a relatively new cultivar, the effect of different storage conditions on ‘Shalimar’ fruit quality is unknown. Therefore, this study examined the fruit quality of 'Shalimar' apples stored under various conditions, including regular atmosphere (RA), CA, and DCA-CD, while also examining the influence of temperature at 1 °C and 3 °C. The main objective is to determine the ideal temperature and storage parameters for achieving long-term preservation of 'Shalimar' apples.

## Materials and methods

The experiment was conducted at the Lake of Constance Research Centre for Fruit Cultivation (KOB), Ravensburg, Germany. 'Shalimar' apples were harvested from the organic field in Eschau (47° 46′ 47.3′′ N and 9° 32′ 05.9′′ E) from KOB in 2019. The trees were planted in 2011 with a spacing of 1 m × 3.5 m, grafted on M9 rootstock. The plot did not have an irrigation system; instead, it relied solely on natural irrigation. Mechanical thinning of the trees was carried out using a Darwin thinning machine. The orchard's location near the Lake of Constance which mitigates the risk of severe frost due to the lake's moderating influence on the weather. The annual temperature and precipitation of the year 2019 was 10.1 °C and 990 mm respectively, which was very close to the long-term average from the year 1961–2019 i.e. 8.5 °C and 957 mm respectively. The plant collection and use were in accordance with all the relevant guidelines. Immediately after harvest, apple were hand-sorted, and apple of similar sizes (65 to 70 mm diameter) were used for the study. The fruit were harvested at the time of their commercial peak in the region, at a firmness of 81 N, total soluble solids (TSS), ° Brix content of 14.0, and starch levels within the range of 4 to 6. After harvest, half of the fruits received 1-MCP treatment, while the remaining half was untreated. Subsequently, the fruits were stored under five distinct storage conditions as outlined in Table 1. For 24 h, the fruit were exposed to 650 µL L^−1^ of 1-MCP solution (SmartFresh™, Agrofresh®, for research 0.014% of active ingredient) in hermetically-closed ventilated chambers. The storage conditions employed for the experiment were: (1) RA at 1 °C; (2) RA + 1-MCP at 1 °C; (3) CA at 1 °C; (4) CA + 1-MCP at 1 °C; (5) RA at 3 °C; (6) RA + 1-MCP at 3 °C; (7) CA at 3 °C; (8) CA + 1-MCP at 3 °C; (9) DCA at variable temperature (~ 2.5 °C); (10) DCA at variable temperature (~ 2.5 °C) + 1-MCP (Table 1). After 7 months of respective storage conditions, fruit were stored for additional 7 days at 20 °C to simulate shelf-life conditions.

**Table 1:** Overview of the treatments and the storage conditions.

Conditions	Storage	Temperature (°C)	O_2_ (kPa)	CO_2_ (kPa)	1-MCP (µL L^−1^)
1	RA	1	20.9	0.04	–
2	RA	1	20.9	0.04	650
3	CA	1	1.0	2.5	–
4	CA	1	1.0	2.5	650
5	RA	3	20.9	0.04	–
6	RA	3	20.9	0.04	650
7	CA	3	1.0	2.5	–
8	CA	3	1.0	2.5	650
9	DCA-CD	~ 2.5	~ 0.46	1.2	–
10	DCA-CD	~ 2.5	~ 0.46	1.2	650

Afterwards, the fruit were stored in the RA, CA, and DCA-CD rooms. The initial reduction of *p*O_2_ in CA and DCA was achieved by flushing nitrogen in the rooms. The final *p*O_2_ and *p*CO_2_ were obtained by fruit respiration in the storage facilities. 

The fruit stored in DCA-CD were exposed to variable temperature and *p*O_2_ conditions after identifying the anaerobic compensation point (ACP) by measuring the CO_2_ production rate of the fruit, as designed by Thewes et al.^15^. In practice, the *p*CO_2_ in the storage room is measured, and the value is recorded. The fruit is stored for approximately 12 h without CO_2_ scrubbing. Subsequently, *p*CO_2_ measurements were repeated. The *p*CO_2_ measurements taken before and after the 12-h period were used to determine the ACP value of the fruit, which, in turn, was used to establish the *p*O_2_ and temperature setpoints.

**Figure 1:**
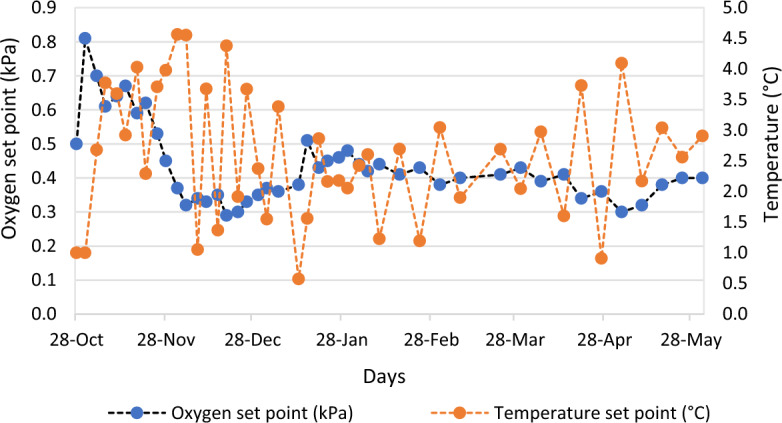
The figure illustrates the variations in oxygen levels (represented in blue) and temperature fluctuations (represented in orange) for DCA-CD during storage.

During the initial 5 months of storage, measurements were repeated at 4-day intervals, while during the following 2 months of storage, measurements were conducted once a week. The average temperature and *p*O_2_ levels for DCA-CD were recorded as 2.5 °C and 0.46 kPa, respectively, as observed in Fig. 1.

Eight fruit per treatment in replicates of three was assessed for fruit firmness utilizing a digital penetrometer (Guess Fruit Texture Analyzer, South Africa) equipped with an 11 mm stamp tip that penetrated to a depth of 1 cm. The analysis was made after removing the skin (1 mm depth) on the equatorial region between the sunny and the shady side; the values were obtained in Newton. For the analysis of TSS and TA, eight fruit per replicate of three were processed into juice. TSS was measured using PR-α32, a refractometer (Brix 0 ~ 32%) from ATAGO (Japan) and expressed as °Brix. TA was determined by titration (815 robotic USB, Metrohm, USA) with 0.1 N NaOH of a solution with 10 mL of juice diluted in 50 mL of distilled water until pH 8.1 was achieved; data were expressed as in mEq 100 ml^−1^. The background color of the fruit was measured using a chromameter (model CR 310, Minolta, Japan) and is expressed as in hue.

Ethylene production and respiration rates were assessed over a 7-day simulated shelf-life period at 20 °C. Four whole fruits per replicates of three were placed into 4.25-L jars, ensuring a continuous flow of air. Respiration rates were determined using a CO_2_ infrared analyzer (Hartmann and Braun GmbH, Germany) that measured the difference in CO_2_ levels between the air injected into the jars and the air exiting the jars, with measurements taken daily throughout the duration of the shelf-life simulation.

For ethylene production measurements, the jars were hermetically sealed for 120 min. Subsequently, approximately 1 mL gas sample was extracted from the headspace of the jars and injected into a gas chromatograph (Carlo Erba Fractovap Series 2150) equipped with a flame ionization detector and a stainless steel 60-mesh column measuring 0.9 m by 1/8″. The injection and oven temperatures were set at 175 °C and 100 °C, respectively. Ethylene levels were quantified on the 3rd, 5th, and 7th days of the shelf-life simulation and are expressed as µL kg^−1^ h^−1^.

### Volatile organic compounds (VOC)

Volatile profile analysis was performed using the solid phase microextraction (SPME) technique applied to the sample headspace. The SPME fiber divinylbenzene/carboxen/polydimethylsiloxane coated (DVB/Car/PDMS, 24 ga, Sigma-Aldrich, Supelco, USA) was used. To conduct this analysis, 10 mL of apple fruit juice was extracted from 8 apples per treatment in replicates of three, which were previously chilled to 1 °C after shelf life and placed in 20 mL vials. For enzymatic activity inhibition, 3.0 g of NaCl were added to the vials. As an internal reference, 10 µL of a 3-octanol solution (1 µL of 3-octanol in 10 mL of distilled water) was added. The VOC samples were extracted from the vials’ headspace using an SPME fiber for 60 min at 35 °C, under stir bar agitation. Previous the fiber exposition the vial`s temperature was equilibrated at the same extraction temperature for 5 min.

The volatile profile analysis was carried out using a gas chromatograph coupled to a mass spectrometer instrument (GC–MS QP2010 Plus, Shimadzu) and equipped with a Zebron_ZB-Wax column (30 m × 0.25 µm i.d.; 0.25 µm of thickness film). The VOCs were thermally desorbed from SPME fiber into the injection port operating in splitless mode at a temperature of 250 °C. Helium was used as the carrier gas at a flow rate of 1.2 mL min^−1^. The column oven temperature remained constant at 35 °C for 3 min, afterward it increased at a rate of 2 °C min^1^ until reaching 80 °C, followed by a further increase at 5 °C min^−1^ until reaching 230 °C, which was then maintained for 5 min. The GC/MS interface and ionization source were both set constantly at 230 °C. The ionization source was set at 70 eV and single quadrupoles mass analyzer operated in full scan mode with a mass range of 35 to 350 m/z. Before conducting the sample analysis, a homologous alkane series was run to determine the linear retention index. The identification of individual volatile organic compounds (VOCs) was achieved by comparing their experimental mass spectra with those found on National Institute of Standards and Technology (NIST) spectra library, and by comparison de experimental LRIs with those provided on the NIST. The concentrations of the individual compounds were calculated by comparing their measured areas with those areas from the internal standard 3-octanol, assuming a response factor between them was one, as described by Both et al.^29^.

### Statistical analysis

This study was conducted in a completely randomized design. Analysis of variance was carried out to identify any difference between treatments (*p* < 0.05). Data that showed significant differences were subjected to Tukey’s Test (*p* < 0.05). To visualize the effects of different storage conditions on fruit quality and volatile compounds, parameters were subjected to a principal component analysis (PCA). Before this analysis the data matrix was auto-scaled to define the same weight for all variables (mean = 0, variance = 1). Statistical analysis was performed using SISVAR software^30^.

## Results and discussion

**Figure 2:**
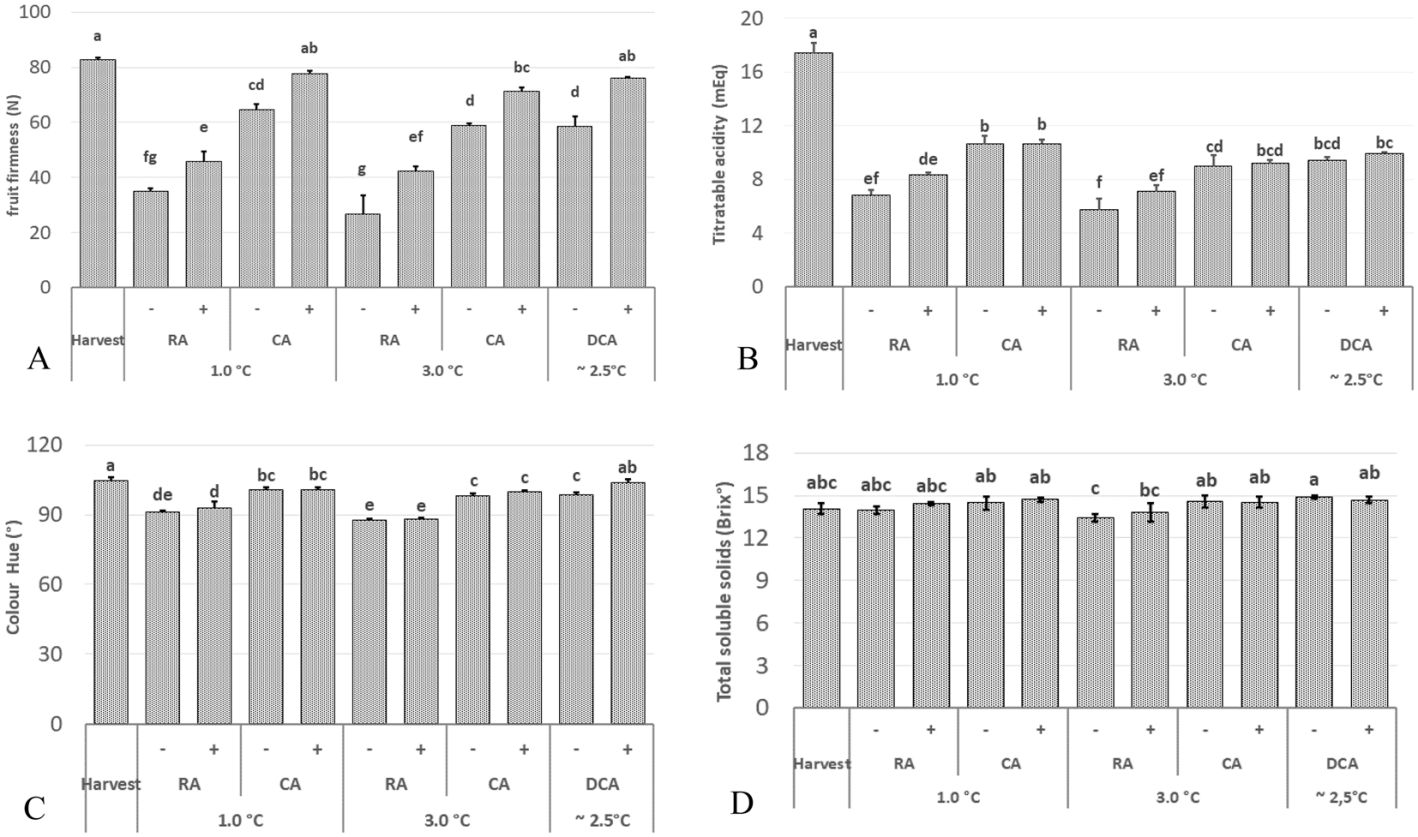
(**A**) Firmness (N); (**B**) Titratable acidity (mEq); (**C**) Background color (Hue°); (**D**) Total soluble solids (Brix °) after 7 months of storage + 7 days of shelf-life) at different storages for 'Shalimar' variety, where ‘ + ’ denotes treatment with 1-MCP and ‘−’ denotes untreated fruit. Mean values with the same letter show no significant difference (Tukey test, *p* ≤ 0.05).

Lowering oxygen levels during storage has been shown to improve the quality retention of apples by reducing the fruit's metabolic activity^18,19,31^. This metabolic process involves various pathways that lead to the production of carbon dioxide through glycolysis and the tricarboxylic acid cycle, as well as the consumption of oxygen through the mitochondrial electron transport chain^32^. Consequently, storage conditions characterized by reduced oxygen and increased carbon dioxide levels are associated with better quality maintenance for most apple varieties. Our study yielded similar results, as apples stored under CA, and DCA, maintained higher firmness, TA, and green background color compared to those stored under RA conditions (Fig. 2).

The application of 1-MCP is an effective technique to enhance firmness and extend the storage life of apples. This effect is achieved by competitively binding to the ethylene receptors in plant tissues, thereby inhibiting ethylene action Consequently, 1-MCP treatment results in reduced respiration rates, delayed fruit softening, the preservation of TA, and the retention of the green background color, which consumers associate with freshness. In our study, apples treated with 1-MCP consistently maintained higher firmness across all storage conditions compared to untreated fruit (Fig. 2A). Notably, apples stored under CA at 1 °C and DCA with 1-MCP maintained a firmness level of more than 75 N, which was remarkably similar to the firmness at the time of harvest. It is worth noting that Hoehn et al.^33^ suggested that the minimum acceptable firmness for consumers is 40 N. This preference is likely due to the perception that firmer fruits are fresher and less susceptible to fungal decay. As a result, for the ‘Shalimar’ apple variety, it may not be necessary to treat apples with 1-MCP if stored under CA and DCA conditions, as fruit stored in DCA untreated with 1-MCP maintained firmness higher than 40N^33^. However, for RA storage, 1-MCP treatment can be essential to maintain the desired firmness level as preferred by consumers.

Moreover, combining 1-MCP treatment with CA and DCA storage strategies may offer enhanced economic benefits for storage operators catering to specific consumer preferences. Consequently, our findings suggest that the use of 650 µL L^−1^ of 1-MCP is an effective method for preserving firmness in 'Shalimar' apples. Additionally, the green background color is a critical quality attribute influencing consumer purchasing decisions. It conveys the impression of freshly harvested fruit^34^. During storage, there was observed a shift from green to yellow background color, led by degradation of chlorophyll, which is consistent with previous findings^1^. In our study, apples subjected to DCA storage and treated with 1-MCP exhibited a hue angle of 103.5°, which was the highest among all treatments.

It may be inferred that 'Shalimar' apples stored under DCA conditions with 1-MCP treatment are the most preferred by consumers in terms of color, followed by DCA and CA at both 1 °C and 3 °C. On the other hand, the Hue ° values recorded for RA-stored apples at 1 °C and 3 °C were 91.2° and 87.8°, respectively. This suggests that fruit stored in regular atmosphere showed more advanced senescence, as indicated by Watkins^6^. Furthermore, Sisler^35^ found a positive correlation between yellow coloration and fruit softening, a similar trend was observed in this study (Fig. 2A,C). Although, it is worth noting that 1-MCP treatment did not significantly affect the background color of apples during storage in RA and CA-stored fruit, which aligns with the findings of Dauny and Joyce^36^. However, DCA with 1-MCP showed a greener color compared to DCA without 1-MCP, indicating DCA storage improves the efficacy of 1-MCP in the maintaining background color of ‘Shalimar’ fruit during storage.

Storage temperature plays a crucial role in maintaining fruit quality during storage. Studies recommend specific storage temperatures for different apple varieties. For instance, ‘Braeburn’ apples are recommended to be stored in CA conditions at temperatures between 0.5 and 1.5 °C, while 'Galaxy' apples can be stored at 2.0 °C to 2.5 °C. Mccormick et al.^23^ suggest that storing fruits at 4 °C instead of 1 °C can result in a 35% energy savings. Weber et al.^18^ found that ‘Braeburn’ apples can be stored at 3 °C in CA and DCA conditions without significant differences in fruit quality compared to storage at 1 °C. Similarly, in this study, the storage temperature did not have a significant effect on background color after 7 months of storage and 7 days of shelf life at 20 °C for the 'Shalimar' apple variety as observed no significant differences in firmness, background color, TA, and TSS between apples stored at 1 °C and 3 °C under CA/ULO and DCA conditions. This is particularly is a remarkable finding considering the rising energy costs in recent years^18^.

Ethylene, an essential plant hormone, plays a pivotal role in various plant and fruit developmental processes, such as germination and optimal fruit ripening. However, excessive ethylene production during storage is associated with accelerated fruit ripening and senescence^23,37^. Ethylene synthesis increases the rate of fruit respiration, which is directly related to fruit quality deterioration during storage^3^. Consequently, measures are taken to minimize ethylene levels during storage.

**Figure 3:**
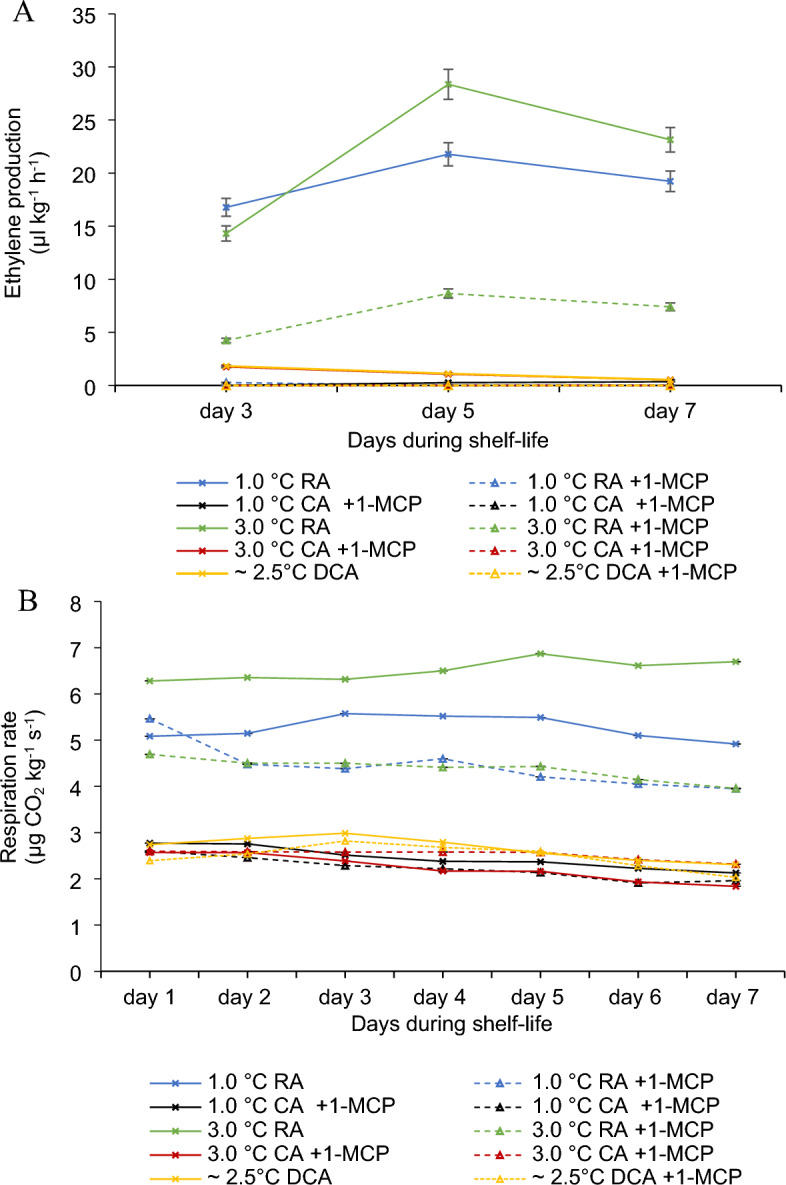
Respiration rate (**A**) and ethylene production (**B**) for ‘Shalimar’ apples at different storages 7 months and during shelf-life at 20 °C.

Limiting oxygen concentration and lowering the temperature during storage are common techniques to reduce both respiration and ethylene biosynthesis and action^29^. In our study, apples stored in CA and DCA-CD showed reduced ethylene production during shelf-life compared to those stored in RA conditions (Fig. 3A). Additionally, the respiration rate was significantly higher in RA-stored apples, with or without 1-MCP treatment, compared to those stored in CA and DCA atmospheres (Fig. 3B). This demonstrates the positive correlation between respiration and ethylene, confirming previous studies^9,19,38^. Furthermore, the role of 1-MCP in inhibiting respiration and, consequently, ethylene production was verified, aligning with the results of Sisler^35^ and Weber et al.^19^.

According to Sisler^35^, 1-MCP blocks ethylene receptors, preventing ethylene binding and the subsequent ethylene-induced responses. This was also evident in our results, as RA-stored apples treated with 1-MCP exhibited lower ethylene production and respiration than untreated apples (Fig. 3A). However, CA and DCA-stored apples, with or without 1-MCP treatment, did not show significant differences in ethylene production and respiration during shelf.

**Figure 4:**
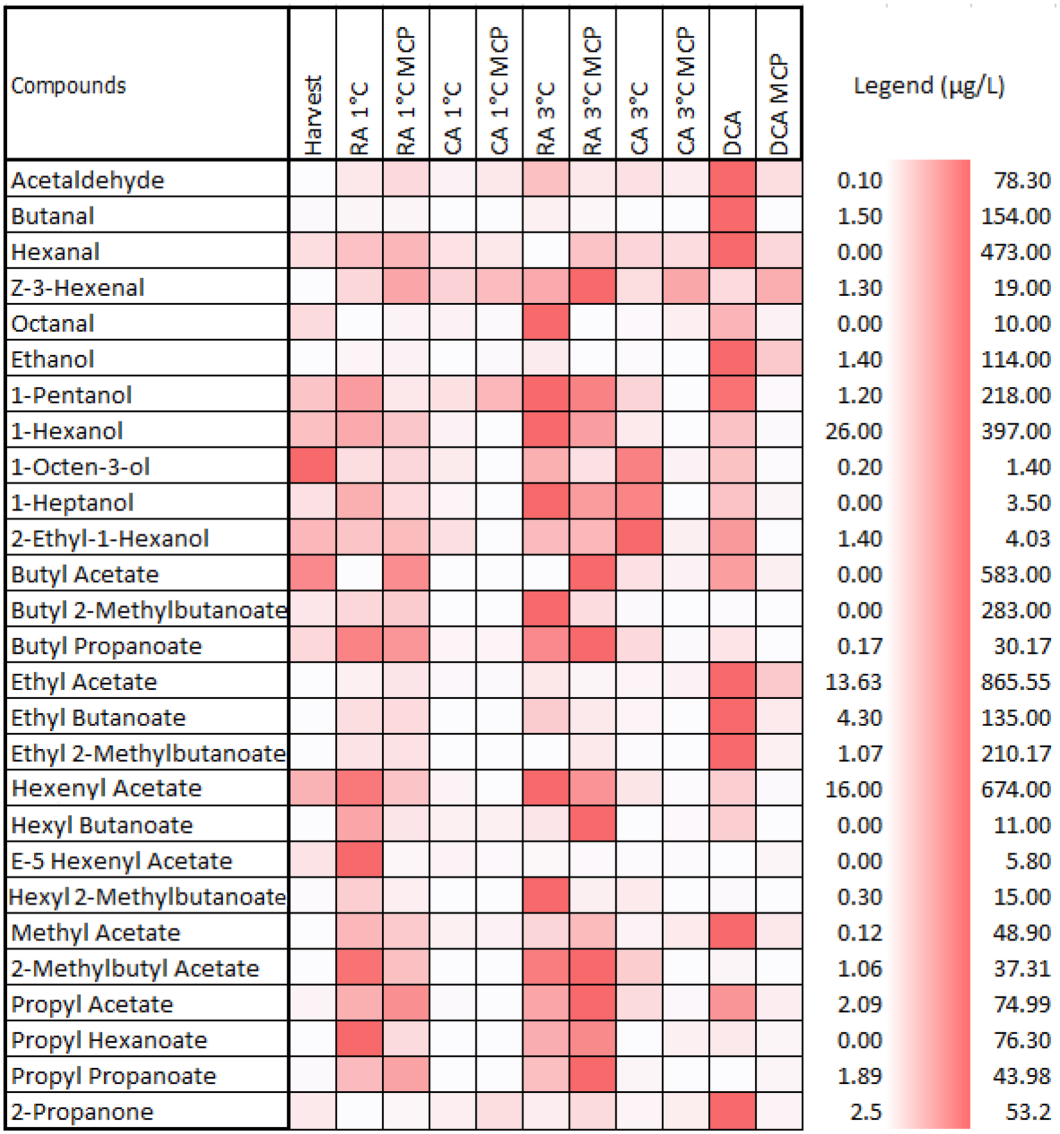
Volatile compounds at different storage techniques after 7 months of storage plus 7 days shelf-life at 20 °C. The color scale illustrates the scaled abundance of each compound, where a lighter shade indicates a lower abundance, while a darker shade signifies a higher abundance of the compound. The legend provides information on the minimum and maximum values for each compound in µg/l.

Esters are the main VOC that confer apples a fruity aroma^39^. Among all esters, butyl acetate, 2-methyl butyl acetate and hexyl acetate contribute the most to apple aroma^40^. In Fig. 4 shows a high concentration of butyl acetate at harvest, followed by fruit stored in regular atmosphere. It is noteworthy that the concentration of butyl acetate was higher in the fruit stored under a DCA when compared to the CA. Similar results were observed for 2-methyl butyl acetate and hexyl acetate.

Methyl acetate concentration was observed higher in the fruit stored in DCA; followed by regular atmosphere and a CA. The presence of methyl acetate is associated with flesh breakdown^41^. However, it was not verified in our results, as this association is maybe variety-dependent. The presence of butyl propanoate, 5-hexenyl acetate, hexyl butanoate and hexyl 2-methyl butanoate were observed in higher concentrations for the fruits stored in regular atmosphere (Fig. 4). These results are in line with previous studies Bangerth et al.^26^ and Weber et al.^19^ which report an increment of the ester with advancement in the ripening process. The general pattern showed that fruits treated with 1-MCP had lower ester concentrations when compared to untreated fruits. The most abundant alcohol present in 'Shalimar' after storage was ethanol and 1-hexanol. The highest concentration of ethanol was observed in DCA. The recent studies by Weber et al.^42^ show a positive correlation between ethanol and flesh firmness until 472 µL L^−1^ and a decrease in ethylene biosynthesis until 500 µL L^−1^. This explains the lower ethylene production during the 7 days shelf-life storage for the fruit stored in DCA (Figs. 3A, 2A). The high ethanol concentration also explains the presence of ethyl esters derived from ethanol, like ethyl butanoate, ethyl 2-methyl butanoate, and others^42^.

The use of 1-MCP has an adverse effect on production of alcohol compounds affecting the ester formation^19,41^. This can also be verified from our findings (Fig. 4). The highest concentration of hexanal was observed in the fruits stored in DCA. On the other hand, hexanal was not detected in apples stored under regular atmosphere at 3 °C. Hexanal is known to be a natural molecule characterizing apple aroma. Furthermore, the presence of hexanal is linked to considerably reducing the growth potential of inoculated yeast^43^, which might also indicate longer shelf life.

### Principal component analysis (PCA)

**Figure 5:**
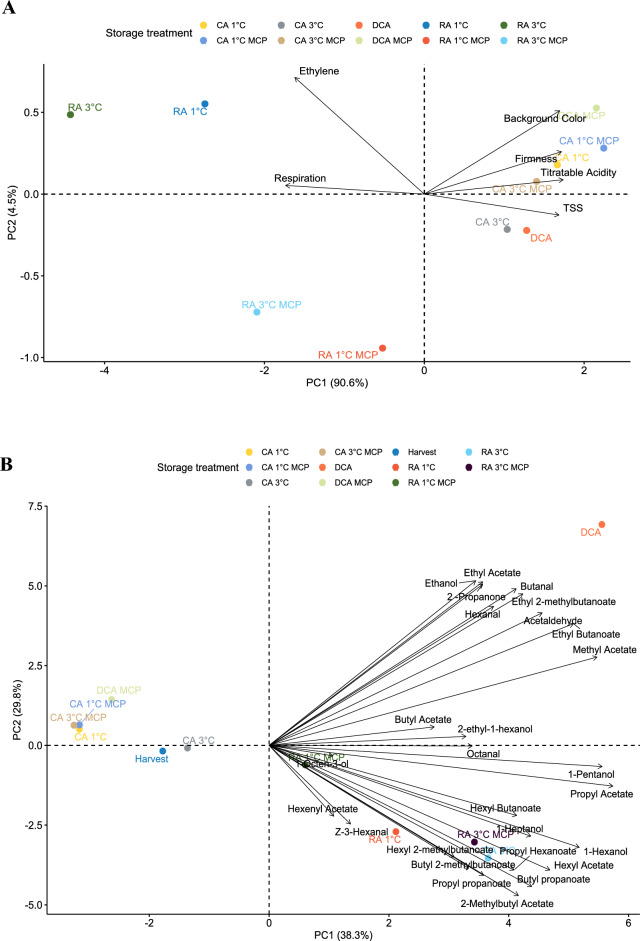
Principal component analysis (PCA) for quality parameters (**A**) and volatile compounds (**B**) for ‘Shalimar’ apple after 7 months plus 7 days of shelf-life at 20 °C.

To understand the relationships between the storage conditions and the determined variables in this experiment, two PCAs were conducted. Figure 5A shows the relationship between different storage conditions and fruit quality parameters. In Fig. 5A, the principal component one (PCI) and two (PCII) together explained 95% of the overall variable variation, whereas in Fig. 5B, PCI and PCII together explained 68% depicts the relationship between storage conditions and different volatile compounds.

The primary aromatic compounds like 2-methyl butyl acetate, hexyl acetate and ethyl butanoate were highly associated with RA and DCA in the 2nd and 4th quadrants, respectively (Fig. 5B). Fruit stored under regular atmosphere were associated with ethylene production andrespiration rate. These results indicate that RA-stored fruit were more mature and had lower quality than fruit stored under CA at 1 °C and DCA with and without 1-MCP (Fig. 5A).

Fruit stored with CA at 1 and 3 °C and DCA with 1-MCP were highly associated with TSS and acidity, and were inversely associated with traits that are usually observed in fruit closer to senescence; for example, higher ethylene, respiration. On the other hand, CA 1 and 3 °C and DCA with 1-MCP shows lower association to volatile compounds (Fig. 5B). At the same time, DCA is a sole treatment that shows a higher association with positive quality traits: total soluble solid, firmness, color (Fig. 5A) and a higher association with volatile compounds such as ethyl-2-methyl butanoate, ethyl acetate, and 2-propanone (Fig. 5B). CA and DCA-stored fruit do not exhibit high differences between 1-MCP treated and untreated fruit (Fig. 5). However, fruit stored in regular atmosphere at 3 °C with 1-MCP loosely correlates with ethylene and respiration (Fig. 5A).

In contrast, untreated fruit depicts a higher association with respiration and ethylene production during shelf-life storage. Overall, it can be concluded that the use of 1-MCP does not pointedly affect the quality parameters of the 'Shalimar' apple variety after 7 months and 7 days of storage at 20 °C, especially for CA and DCA-stored fruit. However, in Fig. 5B, it can be observed that CA and DCA fruit treated with 1-MCP shows a lower correlation to volatile compounds. Similar results were observed by Lurie et al. (2002), where the 'Anna' apple treated with 1-MCP had less fruity and overall aroma. Therefore, in this study, the use of 1-MCP suppresses the production of volatile compounds for the 'Shalimar' apple in long-term storage.

CA at 1 °C and 3 °C and DCA with and without 1-MCP are promising techniques for maintaining the fruit quality of the 'Shalimar' apple. However, DCA without 1-MCP shows a higher correlation with volatile compounds and quality parameters such as high firmness, TSS and TA.

Summarizing, the fruit stored in regular atmosphere at 1 °C and 3 °C with and without 1-MCP showed a high concentration of volatile compounds and high TSS but was unable to retain firmness, TA, and green background color as compared to other treatments. The fruit showed higher respiration and ethylene production during the shelf-life storage. Therefore, we can conclude that regular atmosphere at 1 °C and 3 °C with and without 1-MCP is not suitable for the 7-month and 7 days at 20 °C storage of the 'Shalimar' apple. The fruit stored in CA at 1 °C with and without 1-MCP maintained higher firmness, TSS, and TA and inhibited ethylene production during the days of shelf-life at 20 °C. Therefore, we can conclude that a CA at 1 °C is an efficient way for long-term storage of the 'Shalimar' apple. However, the fruit will be more likely to have less apple aroma when stored in a CA at 1 °C with and without 1-MCP, which may affect consumer preference.

The fruit stored in CA at 3 °C did not show significant differences in major quality parameters like firmness, TA and TSS compared to CA at 1 °C. 'Shalimar' apple fruit can be stored at 3 °C when in CA storage without any significant changes when compared to storage temperature of 1 °C. DCA at variable temperature (~ 2.5 °C) showed the highest occurrence of healthy fruit among all other treatments. DCA was efficient in inhibiting ethylene production during the 7-day shelf-life at 20 °C, thereby maintaining quality parameters like firmness, TA and TSS. These findings can make an impact on saving energy in storage. Fruit are stored at low temperatures to ensure higher quality after storage. The optimal scenario would involve storing the fruit at the highest feasible temperature without any compromise in fruit quality for each specific variety. In that case, there is a possibility of saving a high amount of energy used for storage every year^18^. Therefore, there is a need to study the optimum temperature at which a specific apple variety can be stored without any loss in quality and the exact numbers on how much energy can be saved in this process. Nevertheless, applying DCA-CD can potentially ensure a higher percentage of healthy fruit after storage in an energy-efficient way.

The use of 1-MCP maintains higher firmness. However, it was evident that fruit stored under DCA without 1-MCP showed higher concentrations of volatile compounds like butyl acetate, 2-methyl butyl acetate and hexyl acetate that contributed to the apple aroma. The use of 1-MCP showed higher firmness in all treatments. Moreover, fruit treated with 1-MCP showed less ethylene production during the shelf-life at 20 °C. The results show the fruit stored under DCA-CD treatment alone was sufficient to obtain optimal quality parameters like firmness, TSS, and higher volatile compounds after 7 months and 7 days of shelf-life at 20 °C.

Regular atmosphere at 1 °C and 3 °C with and without 1-MCP were not suitable for 7 months, and shelf-life at 20 °C for the 'Shalimar' apple to maintain optimal quality. CA at 1 °C and CA at 3 °C with and without 1-MCP were efficient in maintaining the optimal fruit quality after 7 months of storage. However, the fruit stored in CA at 1 °C presented fewer volatile organic compounds. DCA-CD with and without 1-MCP efficiently maintains the 'Shalimar' apple for 7 months storage plus 7 days of shelf-life at 20 °C. DCA-CD without 1-MCP shows high fruit quality and high content of volatile compounds after storage, while the use of 1-MCP was negatively correlated to the aroma compounds. Therefore, it can be concluded that using 1-MCP was effective in maintaining the firmness, TSS, TA and background color of 'Shalimar' apple for 7 months of storage. Furthermore, the storage of fruit in either DCA or CA alone effectively maintains minimal ethylene production and respiration rates during shelf-life, even after seven months of storage. These findings will support the expansion of production, marketing, and consumption of 'Shalimar' apples. Given this cultivar's inherent resistance to apple scab and powdery mildew, it represents a significant step towards sustainable fruit production, reducing reliance on chemical pesticides and contributing to more environmentally friendly farming practices.

## Conclusion

Regular atmosphere at 1 °C and 3 °C with and without 1-MCP were not suitable for 7 months, and shelf-life at 20 °C for the 'Shalimar' apple to maintain optimal quality. CA / ULO at 1 °C and CA / ULO at 3 °C with and without 1-MCP were efficient in maintaining the optimal fruit quality after 7 months of storage. However, the fruit stored in CA at 1 °C presented fewer volatile organic compounds. DCA-CD with and without 1-MCP efficiently maintains the 'Shalimar' apple for 7 months storage plus 7 days of shelf-life at 20 °C. DCA-CD without 1-MCP shows high fruit quality and high content of volatile compounds after storage, while the use of 1-MCP was negatively correlated to the aroma compounds. Therefore, it can be concluded that using 1-MCP was effective in maintaining the firmness, TSS, TA and background color of ‘Shalimar’ apple for 7 months of storage. Furthermore, the storage of fruit in either DCA or CA/ULO alone effectively maintains minimal ethylene production and respiration rates during shelf-life, even after seven months of storage. These findings will support the expansion of production, marketing, and consumption of 'Shalimar' apple. Given this cultivar's inherent resistance to apple scabs and powdery mildew, it represents a significant step towards sustainable fruit production, reducing reliance on chemical pesticides and contributing to more environmentally friendly farming practices.

### Supplementary Information

Supplementary Information 1.

Supplementary Information 2.

## Data Availability

All data generated or analysed during this study are included in this published article [and its supplementary information files].
