# Supplementary material for: Impact of different storage conditions with combined use of ethylene blocker on ‘Shalimar’ apple variety
Source: Sci Rep. 2024 Apr 11;14:8485. doi: 10.1038/s41598-024-57688-6 (PMC11009402; doi:10.1038/s41598-024-57688-6)
Supplement: Supplementary file 2 — Supplementary Information 2. [file 41598_2024_57688_MOESM2_ESM.pdf]

Fruit Quality Parameters

Treatment	Background color	Fruit Firmness	TSS	Titratable acidity
RA 1°C	90.71	3.5	13.7	6.5
RA 1°C	90.99	3.71	14.2	7.2
RA 1°C	91.96	3.45	14.1	6.9
RA 1°C MCP	92.01	4.49	14.6	8.2
RA 1°C MCP	95.77	5.09	14.3	8.4
RA 1°C MCP	90.36	4.41	14.4	8.5
CA 1°C	99.66	6.39	14.6	9.9
CA 1°C	100.87	6.58	14.9	11.1
CA 1°C	101.57	6.78	14	10.9
CA 1° MCP	101.52	7.8	14.9	11
CA 1° MCP	100.89	7.87	14.6	10.7
CA 1° MCP	99.42	8.04	14.7	10.3
RA 3°C	88.35	2.78	14.2	6.6
RA 3°C	87.33	3.38	13.1	5.7
RA 3°C	87.73	2.04	13	4.9
RA 3°C MCP	88.07	4.34	13.3	6.7
RA 3°C MCP	88.42	4.11	14.1	6.9
RA 3°C MCP	88.24	4.45	14	7.7
CA 3°C	97.17	5.97	14.4	8.5
CA 3°C	97.78	5.97	14.5	8.5
CA 3°C	99.21	6.1	14.9	10
CA 3°C MCP	100.43	7.45	14.1	9.3
CA 3°C MCP	99.44	7.17	14.7	9.4
CA 3°C MCP	99.19	7.14	14.8	8.9
DCA	99.54	5.88	14.8	9.3
DCA	97.95	6.38	14.8	9.7
DCA	97.74	5.62	15	9.4
DCA MCP	103.02	7.78	14.7	10
DCA MCP	105.65	7.78	14.5	10
DCA MCP	102	7.74	14.9	9.9

Fruit Respiration

Treatment	Respiration Day 1	Respiration Day 2	Respiration Day 3	Respiration Day 4	Respiration Day 5	Respiration Day 6	Respiration Day 7
RA 1°C	5.15827	5.15827	5.577995	5.86167	6.060597	5.57799465	5.545189
RA 1°C	4.90866	5.111151	5.524657	5.294463	5.294463	4.8191575	4.56126
RA 1°C	5.185361	5.168251	5.617474	5.401417	5.116921	4.90371628	4.643459
RA 1°C MCP	4.069072	3.842315	3.616958	3.404195	3.593167	3.37060822	3.348217
RA 1°C MCP	4.373111	4.141185	4.114645	5.197446	4.317236	4.31723602	4.061563
RA 1°C MCP	7.948151	5.45016	5.414777	5.189161	4.706969	4.46808489	4.438599
CA 1°C	2.786234	2.777609	2.506613	2.293113	2.285926	2.27873943	2.031461
CA 1°C	2.98923	2.969697	2.739437	2.748506	2.739437	2.52871143	2.495226
CA 1°C	2.534561	2.518106	2.29318	2.091566	2.077853	1.87006793	1.851556
CA 1° MCP	2.506121	2.52154	2.264274	2.271283	2.271283	2.03784655	2.012615
CA 1° MCP	2.687917	2.679331	2.440313	2.440313	2.204156	1.98374072	2.175537
CA 1° MCP	2.615063	2.172303	2.151552	1.942622	1.930172	1.71017499	1.693575
RA 3°C	6.764388	6.721448	6.448215	6.468944	6.886591	6.65703819	6.571158
RA 3°C	6.151029	6.13245	6.039551	6.09529	6.562975	6.29119943	6.233138
RA 3°C	5.922886	6.208963	6.457321	6.932036	7.156821	6.8880321	7.28574
RA 3°C MCP	4.649819	4.375399	4.291543	4.389375	4.347447	3.88982112	3.649239
RA 3°C MCP	4.36094	4.079572	4.250558	3.839597	3.802803	3.56512819	3.76601
RA 3°C MCP	5.064897	5.049598	4.957805	5.003702	5.141392	4.98840281	4.448255
CA 3°C	2.764392	2.764392	2.501648	2.053426	2.040181	1.8134939	1.789947
CA 3°C	2.743386	2.734972	2.476208	2.258109	2.251098	2.02598829	1.778442
CA 3°C	2.202018	2.202018	2.180984	2.195007	2.195007	1.95657547	1.943955
CA 3°C MCP	2.633835	2.616901	2.599966	2.608433	2.591499	2.58303179	2.55763
CA 3°C MCP	2.634987	2.62635	2.609075	2.617712	2.600437	2.37581624	2.138239
CA 3°C MCP	2.515821	2.515821	2.524494	2.515821	2.524494	2.2823175	2.258466
DCA	2.867907	3.059107	3.246106	3.06891	2.605287	2.59688451	2.357371
DCA	2.603778	2.595407	2.766351	2.578666	2.570295	2.34843117	2.325412
DCA	2.747385	2.966887	2.947994	2.729945	2.486463	2.253154	2.23862
DCA MCP	2.449399	2.672072	3.09698	2.885256	2.654554	2.42531195	2.175632
DCA MCP	2.305799	2.291911	2.482911	2.27108	2.257192	2.03772238	1.794644
DCA MCP	2.426191	2.652736	2.876358	2.885126	2.86759	2.38965857	2.12439

Volatile compounds

Treatment	Acetaldehyde	2 - Propanone	Methyl Acetate	Butanal	Ethyl Acetate	Ethanol	Propyl Acetate
RA 1°C	7.896061	0	25.35972	11.18687	87.17072	14.0195783	57.52034
RA 1°C	9.58774	0	34.83009	9.797124	130.4541	26.9582668	68.38319
RA 1°C	3.951643	0	9.801381	6.974473	42.52438	0.11682593	36.22145
RA 1°C MCP	21.24953	0	31.22187	15.36532	174.0925	9.14166636	111.3643
RA 1°C MCP	5.320204	1.488736	8.476628	7.050151	110.7129	1.48317029	50.62866
RA 1°C MCP	10.81589	3.31536	10.89125	5.072311	166.44	38.9550846	62.97769
CA 1°C	0	2.972331	4.061833	0.586407	74.59646	1.76132204	3.172741
CA 1°C	4.966793	3.975889	5.043456	3.80525	6.518604	0.0967206	1.11409
CA 1°C	6.133449	4.52552	4.163587	3.26208	55.93321	2.27008432	8.980617
CA 1° MCP	9.245777	8.2444	3.517339	0	8.795296	5.37681857	0.322011
CA 1° MCP	6.226391	12.45524	3.850992	2.365437	2.747713	0.17065843	0.796841
CA 1° MCP	6.19316	2.553038	3.51111	2.192224	83.84977	14.6123159	5.148042
RA 3°C	13.96602	3.719698	13.69059	12.61409	146.8666	2.86007827	59.139
RA 3°C	15.17557	5.052286	9.097766	18.91476	153.5067	31.6867217	69.29255
RA 3°C	34.98205	3.164506	15.50678	10.58111	120.7829	50.0601571	53.69192
RA 3°C MCP	10.89735	11.28389	39.45769	11.38208	78.28274	0.70216737	125.5452
RA 3°C MCP	6.773084	3.061892	5.326085	6.626611	56.60131	4.88976462	101.1963
RA 3°C MCP	4.961008	11.2261	22.37014	6.783371	42.49765	6.15207742	73.75854
CA 3°C	4.798021	2.598822	4.627462	2.550971	82.70703	12.493611	10.81503
CA 3°C	19.07735	0	0	1.930718	48.6478	0	37.52079
CA 3°C	4.767402	3.747985	5.437214	4.056928	64.08668	3.70944351	25.39712
CA 3 MCP	8.27316	6.49804	5.890977	3.190813	84.71852	0	6.13369
CA 3 MCP	4.233321	2.702612	8.159923	0	65.63559	0	5.27224
CA 3 MCP	4.942477	3.143597	5.112098	4.136544	85.42829	4.54587649	6.079365
DCA	99.36938	55.28391	82.82659	16.69708	1396.75	419.89043	107.8967
DCA	35.2455	46.63242	53.66955	306.2798	770.8257	126.604901	57.13535
DCA	19.76419	11.92885	10.45842	139.1849	429.0867	124.284737	48.42811
DCA MCP	10.44199	2.401497	5.921319	3.752931	286.0055	79.1228182	14.20244
DCA MCP	17.10464	3.525547	11.49977	0.496756	425.3424	123.644453	12.11874
DCA MCP	4.33751	1.617991	4.598521	3.070401	232.1262	39.0462541	10.63823

Volatile compounds

Treatment	Ethyl Butanoate	Propyl Propanoate	Ethyl 2-Methylbutanoate	Butyl Acetate	Hexanal	2-Methylbutyl acetate	Octanal
RA 1°C	124.5311	26.79475	32.77704	0	207.8858	48.5216341	0
RA 1°C	153.8259	23.72143	53.64887	0	176.4429	32.6879733	0
RA 1°C	80.30172	10.73922	24.84673	0	190.4344	25.0588114	0
RA 1°C MCP	104.7192	40.09862	31.00651	634.3844	411.7816	16.0258453	0.574334
RA 1°C MCP	81.67489	18.68758	27.42522	297.204	121.2636	12.191423	0.331272
RA 1°C MCP	218.9318	23.78284	57.31271	399.7267	145.6591	20.5887434	0
CA 1°C	8.009749	1.976188	1.590657	0	62.16073	1.22783104	0.475088
CA 1°C	1.334524	0.262785	0.565272	0	77.24203	0.14316268	0
CA 1°C	12.08995	3.427165	2.664322	0	117.6335	1.80129653	0.606006
CA 1° MCP	0.427312	2.340177	0	4.563877	84.1448	3.07350167	0
CA 1° MCP	0	0.714646	0	0	27.61175	0	0
CA 1° MCP	12.1633	2.100681	3.196659	0	88.6475	1.04584483	0.338574
RA 3°C	149.6835	14.36244	0	0	0	27.6786751	6.42557
RA 3°C	262.3054	20.73025	0	0	0	37.0689285	0.4626
RA 3°C	152.1386	23.15287	0	0	0	34.1855342	7.435057
RA 3°C MCP	93.56944	47.10535	34.26811	878.7066	253.7996	68.8559921	0
RA 3°C MCP	90.75307	50.70537	29.00128	296.9191	91.07001	12.1797357	0
RA 3°C MCP	56.36473	34.12968	18.57936	575.186	206.4288	32.6871794	0
CA 3°C	14.22404	2.812186	4.551427	85.56811	116.9898	9.54662132	0
CA 3°C	75.11526	0	0	42.56166	104.7439	14.8471699	0
CA 3°C	25.74784	8.75677	7.74273	198.3342	163.4658	14.8348518	0.425354
CA 3 MCP	8.432471	1.204031	2.283005	43.75624	131.4146	2.05743701	0.73856
CA 3 MCP	6.9717	1.305539	2.332406	33.77299	84.91528	1.02826271	0.288192
CA 3 MCP	11.17669	2.775902	3.357414	46.95349	101.4966	1.17328317	0.387385
DCA	768.4054	4.965139	361.8674	669.1495	1057.76	8.54877039	5.038528
DCA	566.1291	0	138.7607	285.1563	242.739	0	0.918615
DCA	366.7571	0	129.886	173.4373	117.9691	0	1.004025
DCA MCP	60.75113	6.908062	19.49617	41.02535	85.12033	0.73159262	0.275197
DCA MCP	130.6136	1.0991	25.97838	63.41463	140.363	2.88218542	0.462887
DCA MCP	38.39528	3.154538	8.363534	58.93231	140.3877	0	0.3262

Volatile compounds

Treatment	Butyl propanoate	3-Hexanal	Butyl 2-methylbutanoate	1-Pentanol	Hexyl Acetate	Propyl Hexanoate	Hexenyl acetate
RA 1°C	27.45239	5.328934	67.56566	10.97148	632.0602	6.29004962	11.95594
RA 1°C	25.65357	8.44706	55.75396	11.71865	617.8718	5.43091601	23.34815
RA 1°C	22.91798	3.630266	42.87546	9.436157	771.8026	5.3571881	17.41728
RA 1°C MCP	21.21444	9.021672	115.8624	0	411.8371	1.80725549	0
RA 1°C MCP	21.0916	13.3465	39.39826	0	189.7147	0	0
RA 1°C MCP	21.68221	12.93568	58.51341	9.91914	287.5998	2.02684361	2.253838
CA 1°C	4.276166	6.073211	1.041493	4.425773	81.99711	0	1.98619
CA 1°C	1.405052	3.972756	0	3.349644	36.46706	0	0
CA 1°C	0	9.053677	0	4.115523	75.78503	0	1.283252
CA 1° MCP	0	11.84217	0	0.970246	19.25619	0	0
CA 1° MCP	1.114	6.215843	0	21.31416	1.883733	0	0
CA 1° MCP	4.869011	8.983521	0	1.793556	26.13763	0	1.508018
RA 3°C	11.43681	12.89572	243.6907	10.81124	637.7909	1.08121486	0
RA 3°C	20.94499	14.24654	315.801	19.74117	921.7369	0.26949636	0
RA 3°C	39.41967	6.829535	87.12791	16.6511	675.9583	7.85453812	1.783036
RA 3°C MCP	46.39302	30.17596	16.89921	17.27307	588.333	0	0
RA 3°C MCP	18.28896	8.852996	62.80373	11.11153	563.4286	12.4204315	0
RA 3°C MCP	26.86403	18.06122	62.75841	11.42615	463.6887	0.91811875	1.588756
CA 3°C	4.48935	3.401124	16.90444	3.967685	73.95518	0	0.966519
CA 3°C	2.466337	4.487107	0	4.241175	136.7196	0	0
CA 3°C	15.63616	7.024927	0	7.277333	193.4895	0	0
CA 3 MCP	1.960331	15.70005	0	0	31.75877	0	0
CA 3 MCP	0.620511	7.223833	0	1.712554	20.4931	0	0.773457
CA 3 MCP	0	11.78308	0	1.872268	31.26362	1.4201523	0
DCA	10.62476	12.8292	0	20.83785	469.8203	2.22492865	0
DCA	2.709815	3.116959	9.82096	15.99459	142.5311	0	0
DCA	2.48202	0.35217	0	7.743442	109.2224	0	0
DCA MCP	0.517361	7.884665	0	1.957777	22.22257	0.8837155	0
DCA MCP	0	16.02455	0	2.880142	33.66486	0	1.433782
DCA MCP	0	8.490398	0	1.81E-05	43.20205	0	1.388916

Volatile compounds

Treatment	1-Hexanol	Hexyl Butanoate	Hexyl 2-methylbutanoate	1-Octen-3-ol	1-Heptanol	2-Ethyl-1-Hexanol
RA 1°C	316.9358	9.91053	23.4143	0.433671	1.425754	2.26845051
RA 1°C	397.0714	8.389069	16.3627	0.437474	1.486885	1.47527478
RA 1°C	329.1461	1.593114	0	0.537106	2.647592	3.35959964
RA 1°C MCP	343.1823	3.772099	0	0.800677	1.490804	3.47776341
RA 1°C MCP	160.5528	1.466576	0	0.458586	0.632547	2.86515395
RA 1°C MCP	205.5549	0	12.85771	0.360718	0.338031	1.26444026
CA 1°C	85.7957	0	0.724954	0.321506	0	1.15279928
CA 1°C	53.35298	2.932516	0	0.413483	0.592235	3.08658682
CA 1°C	77.68103	0	0.990341	0.30489	0	1.43897834
CA1 MCP	20.99787	2.218201	0	0	0	0.9022056
CA1 MCP	21.33618	0.59049	0	0.351913	0	2.38900249
CA1 MCP	35.93018	0	0.826402	0.149033	0	0.85309846
RA 3°C	466.788	1.481421	38.4129	0.915593	2.782603	2.51337557
RA 3°C	750.0977	3.753035	42.48591	1.072872	4.692197	2.38718726
RA 3°C	578.6294	0	43.98511	0.833546	3.015361	2.73029684
RA 3°C MCP	450.9723	25.68334	0	0.702474	1.987629	2.49950505
RA 3°C MCP	375.9172	7.050856	12.04697	0	2.178887	1.53172963
RA 3°C MCP	362.8108	0.640849	0	0.617404	2.754414	3.83852218
CA 3°C	65.55132	0	1.827453	0.236584	0.166032	1.87020233
CA 3°C	77.38255	0	9.965528	3.509846	7.75905	8.61170349
CA 3°C	154.8838	0	4.425644	0.46227	0.625122	1.61182533
CA 3 MCP	32.98712	0.955467	0	0.375645	0	1.62286555
CA 3 MCP	22.63124	0	1.073062	0.242285	0	1.33409915
CA 3 MCP	29.6474	0	1.437574	0.235649	0	1.84491977
DCA	460.3438	10.3804	0	1.251855	3.373881	5.62931371
DCA	198.1276	0	3.808027	0.520718	0	1.45839832
DCA	101.6243	0	0	0.489923	0.763461	2.38901409
DCA MCP	30.22044	0	0	0.307016	0.161836	1.98534823
DCA MCP	36.08123	0	0.911311	0.263937	0	1.12946889
DCA MCP	49.56457	0	1.120886	0	0.35853	0.95692501

Ethylene

Treatment	Ethylene day 1	Ethylene day 2	Ethylene day 3
RA 1°C	10.64211	15.50666	12.19671
RA 1°C	21.15114	29.08178	27.46734
RA 1°C	18.53625	20.74142	18.02345
RA 1°C MCP	0	0	0
RA 1°C MCP	0.354889	0	0
RA 1°C MCP	0.467257	0	0
CA 1°C	0	0	0
CA 1°C	0	0.78249	1.050067
CA 1°C	0	0	0
CA1 MCP	0	0	0
CA1 MCP	0	0	0
CA1 MCP	0	0	0
RA 3°C	14.89419	30.46944	19.8537
RA 3°C	14.21496	26.18855	26.61149
RA 3°C	13.81893	28.41968	22.96145
RA 3°C MCP	4.437656	8.408106	8.756892
RA 3°C MCP	2.116458	4.289624	3.945977
RA 3°C MCP	6.165704	13.30372	9.514268
CA 3°C	1.97792	0.797837	0
CA 3°C	1.027372	0.709701	0
CA 3°C	2.23885	1.671036	1.594979
CA 3 MCP	0	0	0
CA 3 MCP	0	0	0
CA 3 MCP	0	0	0
DCA	0.944252	0.922685	0
DCA	2.897628	1.579686	1.604311
DCA	1.636762	0.841848	0
DCA MCP	0	0	0
DCA MCP	0	0	0
DCA MCP	0	0	0
